# Bladder metastasis presenting as neck, arm and thorax pain: a case report

**DOI:** 10.1186/s12998-016-0097-8

**Published:** 2016-05-04

**Authors:** Clinton J. Daniels, Pamela J. Wakefield, Glenn A. Bub

**Affiliations:** Chiropractic Clinic, VA St. Louis Healthcare System, 1 Jefferson Barracks Rd, Saint Louis, MO USA; Logan University, College of Chiropractic, 1851 Schoettler Rd, Chesterfield, MO USA; 811 Rowell St., Steilacoom, WA 98388 USA

**Keywords:** Chiropractic, Neck pain, Transitional cell carcinoma, Bladder cancer, Metastasis, Case report

## Abstract

**Background:**

A case of metastatic carcinoma secondary to urothelial carcinoma presenting as musculoskeletal pain is reported. A brief review of urothelial and metastatic carcinoma including clinical presentation, diagnostic testing, treatment and chiropractic considerations is discussed.

**Case presentation:**

This patient presented in November 2014 with progressive neck, thorax and upper extremity pain. Computed tomography revealed a destructive soft tissue mass in the cervical spine and additional lytic lesion of the 1st rib. Prompt referral was made for surgical consultation and medical management.

**Conclusion:**

Distant metastasis is rare, but can present as a musculoskeletal complaint. History of carcinoma should alert the treating chiropractic physician to potential for serious disease processes.

## Background

Urothelial carcinoma (UC), also known as transitional cell carcinoma (TCC), accounts for more than 90 % of all bladder cancers and commonly metastasizes to the pelvic lymph nodes, lungs, liver, bones and adrenals or brain [1, 2]. The spread of bladder cancer is mainly done via the lymphatic system with the most frequent location being pelvic lymph nodes. Bladder cancer is the most common malignant disease of the urinary tract with a higher incidence in older age and more prevalent in men than women [3]. There is a higher prevalence in white persons; however, delayed diagnosis has lead to higher mortality rates in black persons [4]. More than 80 % of skeletal metastases are from carcinomas of the lung, breast and prostate with bladder tumors responsible for just 4 % of all bone metastases [5, 6]. Although uncommon, studies confirm that bone is the preferred site of metastasis (35 %) of UC outside of the pelvis, with the spine being most common site (40 percent of bone metastases) [7]. The cervical spine is only affected in 8 to 20 % of metastatic spine disease cases [8, 9]. The most serious complication of UC is distant metastasis—with higher stage cancer and lymph involvement worsening prognosis and cancer survival rate [10]. The 5-year cancer-specific survival rate of UC is estimated to be 78 % [10, 11].

Neck pain accounts for 24 % of all disorders seen by chiropractors [12]. Although infrequently encountered, malignancy and infiltrative processes are a potential pathological source of neck pain [13]. Cancer is among the most common life threatening conditions presenting to chiropractic treatment facilities, with 58.9 % of chiropractors self-reporting identification of previously undiagnosed carcinomas [14]. A recent systematic review identified more than 60 published cases of diverse cancers recognized by chiropractic physicians [15]. The objective of this case report is to describe a patient presenting for chiropractic care with neck, arm and thorax pain due to metastatic disease secondary to urothelial carcinoma and provide brief review including clinical presentation, diagnosis, and treatment.

## Case presentation

### History

An 81-year-old white male presented to the Saint Louis Veterans Health Affairs chiropractic clinic in November 2014 with a referral for low back pain and he described additional complaints of neck pain, upper thoracic pain, radiating numbness, tingling and pain into the left arm, and pain that radiated into his left lateral and anterior chest wall. Coughing and sneezing significantly increased his pain in all areas of complaint and he denied chest pain and shortness of breath. Relevant medical history included concurrent care for high-grade papillary transitional cell carcinoma (TCC) of the bladder, and presence of a pacemaker. He was a 50-year non-smoker, but with a 30-pack year history of cigarette use.

His bladder cancer was diagnosed approximately one year prior after he presented with gross hematuria. Cystoscopy confirmed the presence of an 8 mm papillary tumor near the right ureteral opening and trigone, and a second cystoscopy identified a 5 mm mass on the left lateral bladder wall. Two prior biopsy and transurethral resections of bladder tumor (TURBT) procedures had been performed. Pathology report revealed high-grade transitional cell carcinoma that invaded subepithelial connective tissue, but was negative for muscular infiltration. Upon presenting for chiropractic care he was in the process of completing a second round of Bacillus Calmette-Guérin (BCG) immunotherapy.

### Diagnosis and management

**Fig. 1:**
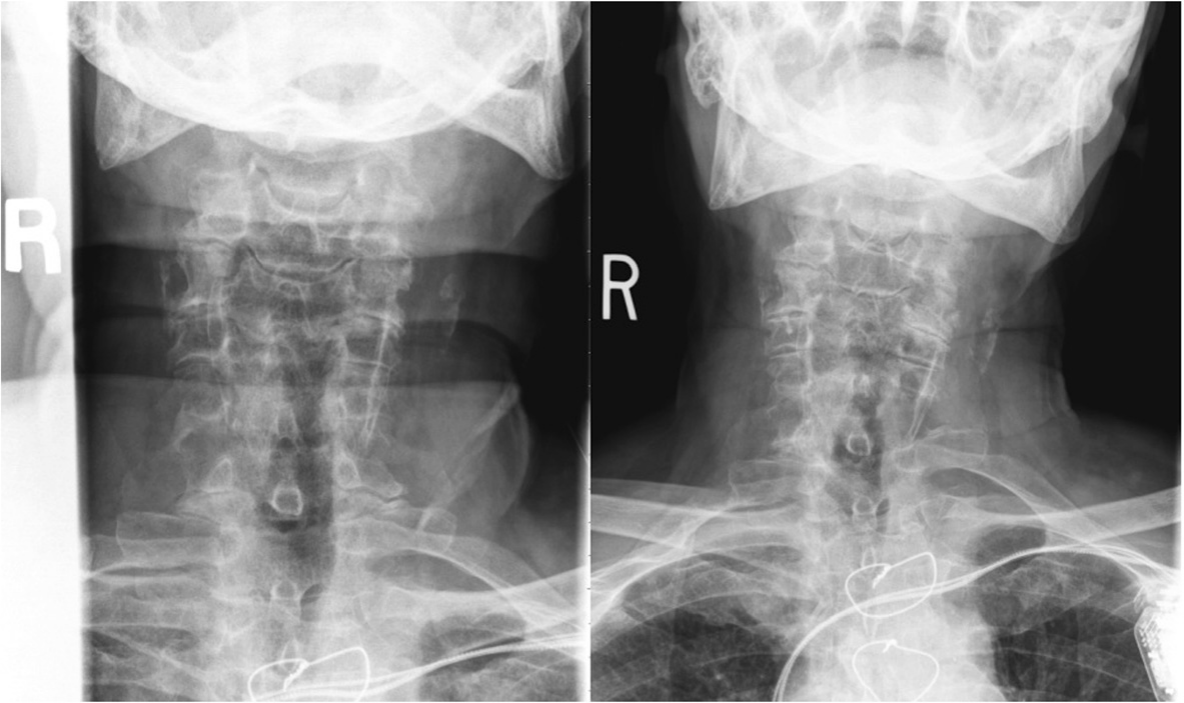
AP cervical radiograph taken in 2010 (*Left*) AP cervical radiograph demonstrating missing left C6 pedicle and articular pillar taken in 2014 (*Right*)

**Fig. 2:**
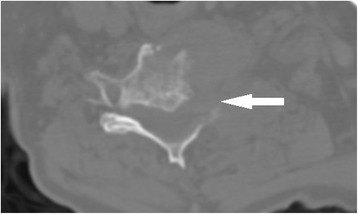
Axial CT demonstrating destructive mass C6 left vertebral body and transverse process

**Fig. 3:**
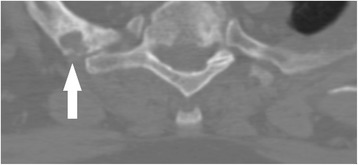
Lytic lesion in right 1st thoracic rib

Cervical radiographs taken one week prior to presentation were read as moderate degenerative changes of the cervical spine with moderate carotid bulb atherosclerosis (Fig. 1). Physical examination revealed tenderness to touch of cervical paraspinal muscles and left upper trapezius, and diminished light touch of the left upper extremity. Left anterior chest wall pain was reproduced with manual over pressure of the left lower cervical spine. The provider could not clearly identify the cause of the upper back, chest and arm pain and did not provide any treatment to the cervical region. MRI was contraindicated due to the presence of an implanted pacemaker. Computed tomography was obtained; multiplanar multidetector CT images revealed a large destructive soft tissue mass along the leftward aspect of C6-T1 with frank osseous destruction of the vertebral bodies and transverse processes (Fig. 2). An additional lytic lesion was identified within the first right rib (Fig. 3). The patient was sent to the emergency department for stabilization and consultation with an orthopedic surgeon. He selected non-operative management and was stabilized with a Miami-J cervical collar. The patient expired 4-weeks following the discovery of these lesions on the CT scan.

## Discussion

Metastatic disease of the cervical spine can present with a variety of clinical signs and symptoms; including mechanical, nonmechanical, and referred pain due to pathologic fracture and/or neurologic dysfunction from cord or nerve root compression. Localized nonmechanical pain is the most common complaint, and is often described as not being related to any activities, progressively worsening, and exacerbated in the evening [16]. For patients with a history of carcinoma and a new onset of nonmechanical pain it is imperative to rule out the presence of metastatic disease [17–19]. Our patient’s history of UC, cigarette use, and new onset of neck pain was a red flag for potential pathologic processes.

There is a clear correlation between smoking and the risk of developing bladder carcinomas [20]. Cigarette smoking is credited as responsible for more than 50 % of cases within the developed world [21], and a four to seven times greater risk than nonsmokers [22, 23]. An additional 5 to 10 % of cases can be linked to occupational exposures, such as aromatic amines used in manufacturing of chemical dyes and pharmaceuticals, and in gas treatment plants [24, 25]. While our patient was a long-term non-smoker his prior smoking may still have been contributory to disease development.

The most common symptom of patients with urothelial carcinomas is painless hematuria. Gross blood throughout urination is suggestive of bladder cancer [20]. Our patient sought medical care in the presence of hematuria and this ultimately lead to his cancer diagnosis. Early stage, local bladder disease, or carcinoma in situ, most commonly presents with urinary frequency, urgency and other signs of bladder irritation [20]. If the lesion is located near the urethra or bladder neck then the patient may experience obstructive symptoms. Signs of obstruction include decreased force or intermittent stream, sensation of incomplete voiding and straining. Pain may be present in the flanks with advanced stages of disease caused by urethral obstruction. Physical pain may also present in abdomen, pelvis, buttock or at distant bone sites [20, 26].

Practitioners should begin investigation of patients with urinary symptoms with a thorough history of cigarette smoking and occupational exposures. Physical examination does not provide much insight for early bladder cancer, however, palpable masses of the kidney and pelvis may be detectable in later stages [20]. Urinalysis with urine microscopy and a urine culture is first line screening to rule out infection and hematuria. If bladder cancer is suspected, then urine cytology and cystoscopy would be indicated. Urine cytology is a noninvasive test that consists of sending urine sample to a laboratory for pathologist assessment to identify and monitor for high-grade tumors. Cytology interpretation is user dependent [27] with a sensitivity for carcinoma in situ of 28 to 100 % [28] and specificity exceeding 90 % [29]. Cystoscopy is an office procedure performed under local anesthesia, and is the mainstay of diagnosis and surveillance [20]. This involves insertion of a hollow tube with a lens (cystoscope) into the urethra and advanced towards the bladder. The cystoscope provides information on tumor location, appearance, and size. Bladder wash cytology is very sensitive for carcinoma in situ and obviates the need for random bladder biopsies. Patients with symptoms of bladder cancer should be evaluated with cystoscopy and bladder wash cytology [30].

Patients with a known history of cancer presenting with persistent neck pain (including mechanical and nonmechanical) should be evaluated for pathologic processes [16]. A history of nocturnal pain further escalates the suspicion of a neoplastic process [16]. Early diagnosis can be aided with basic neurologic screening for spasticity, hyperreflexia, Hoffman sign, and abnormal plantar reflexes [31]. In our case, the patient presented with vague mechanical symptoms and neurological symptoms that quickly progressed between visits—although pathologic reflexes remained absent. Evaluation for metastatic disease includes common laboratory tests such as complete blood count, blood chemistry tests, liver function tests, chest radiography, and CT or MRI [32]. Follow-up bone scan may be performed if in the presence of symptoms indicating potential bone metastasis or elevated alkaline phosphatase levels [20].

Men have a higher incidence of spinal metastases than women, and individuals in the fourth and sixth decade are most likely to be affected [33]. The main mechanisms by which a lesion can metastasize to the spine are dependent on the primary neoplasm and include: direct extension or invasion, hematogenous metastasis, and cerebrospinal fluid (CSF) seeding [16]. Direct extension occurs through primary lesions becoming locally aggressive and extending to involve bony spine. Hematogenous seeding is facilitated by the vast arterial supply of the vertebra and via the valveless venous drainage plexi such as Batson’s plexus. Seeding of a primary lesion through the CSF occurs much less frequently and is most often caused by surgical manipulation of cerebral lesions [34]. Post-mortem biopsy was not performed on this patient, therefore it is not possible to definitively state that his cervical metastasis was a direct result of his urinary carcinoma.

Management of metastatic disease of cervical spine requires a multidisciplinary approach. In general, nonsurgical management of metastatic spine is recommended when tumor involvement has not resulted in spinal instability, neurological involvement, and pain nonresponsive to medical management [35]. Nonoperative management consists of radiotherapy, chemotherapy, hormonal therapy, and high-dose steroid therapy [16]. Radiotherapy is specifically aimed to reduce tumor size and response to therapy varies widely depending on tumor type. To reduce localized intramedually edema, acutely presenting symptoms of cervical epidural spinal cord compression by a neoplasm can be managed with corticosteroids such as Dexamethosone [16]. Chemotherapy may be utilized as an adjuvant therapy to treat the primary tumor(s), as it has no direct effect on spinal instability [16]. Although not a primary method of treatment, bisphosphonate use in the setting of metastatic cervical spine disease is advocated to reduce the incidence of skeletal-related events such as pathologic vertebral fractures and cord compression [16]. Surgery is generally palliative and indicated in cases of neurologic dysfunction, spinal instability, and intractable pain (Table 1). In cases of metastatic tumor the most common surgical intervention is anterior cervical corpectomy with fusion. Laminectomy with fusion is used less frequently as most lesions are located anteriorly. However, posterior decompression and stabilization may be the best treatment option at the craniocervical junction [16]. Detailed discussion of each individual therapy is beyond the scope of this paper and can be found elsewhere [8, 36, 37] (Table 2).

**Table 1:** Indications for neurosurgery in the presence of malignancy [35]

Surgical Indications
• Pain due to mechanical compression of pain producing structures or clear instability
• Symptomatic mechanical compression of neurostructures (neurological deficit)
• Rapidly progressing neurological deficit due to mechanical compression
• Unknown primary tumor with clearly defined metastatic involvement of the spine
• Radioresistant tumor
• Neurological deterioration or increasing pain during or after radiotherapy (should be avoided by a careful evaluation of the tumor potential before irradiation is decided)

**Table 2:** Treatment rationale for non-operative procedures

Treatment	Purpose/Goal
Corticosteroid (i.e., Dexamethosone)	Reduce intramedullary edema and subsequent pressure
Chemotherapy/Hormone Therapy	Treat or manage primary tumor
Irradiation	Reduce tumor size
Bisphosphonates	Prevent and/or reduce likelihood of skeletal-related events

The effectiveness of these treatment modalities and the patient survival rate depends on the histological tumor type, tumor stage, therapeutic control of the primary tumor, and spread of the tumor [36]. Indications for treatment are guided not simply by neurocompression, but also by quality of life factors—such as pain and loss of mobility. The oncology clinical decision process is further hampered, as a surgical option is often inappropriate due to possible comorbidities.

One of three cancer patients experience pain either directly related to their lesion or as an adverse result of cancer treatment—for instance radiation related fibrosis and joint contracture or chemotherapy-induced neuropathy [38]. Chiropractic care with high-velocity manipulation is widely considered an absolute contraindication [39]. Potential diminished bone strength and integrity from malignancy puts the patient at risk of skeletal-related events with forceful treatments. Low force treatment techniques such as mechanical-assisted manipulation methods [40], myofascial release, stretching and gentle exercise may be appropriate on a case by case basis as an adjuvant for pain management [39]. While prudent use of chiropractic services in cancer patients may offer effective strategies for reducing the pain and suffering, we do not believe that any chiropractic care was appropriate for our patient’s chief complaint.

## Consent

Written informed consent could not be obtained. The Saint Louis Veterans Health Affairs privacy officer and Research Development Committee provided approval for publication of this report and associated images. A written approval is available for review by the Editor-in-Chief of this journal.

## Conclusions

This case describes the presentation of metastatic urothelial carcinoma as a source of neck, arm and thorax pain. Development of urothelial carcinoma is strongly correlated with smoking and occupational exposures. Although distant metastasis is rare, it can present as musculoskeletal pain and it is not uncommon for previously undiagnosed cases to present to chiropractic physicians.
